# Transformative Approaches to Biomarker Assessment and Clinical Profiles in Pulmonary Hypertension Patients: Short title: Biomarker Assessment and Clinical Profiles in Pulmonary Hypertension Patients

**DOI:** 10.31661/gmj.vi.4132

**Published:** 2025-12-18

**Authors:** Basim M Ali, Furqan Moein Auda, Fatima Abdali Hassooni

**Affiliations:** ^1^ Chemistry Department, College of Science, University of Kufa, Al Najaf, Iraq; ^2^ Faculty of Science, Babylon University, Al Najaf, Iraq

**Keywords:** Pulmonary Hypertension, KCNK3, Homocysteine, Insulin Resistance, Renal Dysfunction, Biomarkers

## Abstract

**Background:**

Pulmonary hypertension (PH) is a progressive disorder marked by elevated pulmonary arterial pressure, leading to right heart failure and high mortality. Emerging evidence links metabolic disturbances, renal dysfunction, and molecular dysregulation to PH pathogenesis. This study aimed to investigate biochemical and molecular biomarkers associated with PH, focusing on renal, metabolic, and vascular parameters.

**Materials and Methods:**

A hospital-based case–control study was conducted at Al-Sadr Teaching Hospital, Iraq, from January to June 2024. Sixty-two PH patients and 30 age- and sex-matched healthy controls were recruited. Fasting venous blood samples were analyzed for urea, creatinine, glucose, insulin, uric acid, troponin I, homocysteine, and KCNK3. Insulin resistance (IR) was estimated using HOMA-IR. Comparisons between groups were performed using independent t-tests, correlations via Pearson’s coefficient, and diagnostic performance assessed using ROC curve analysis.

**Results:**

PH patients exhibited significantly elevated urea, creatinine, glucose, insulin, uric acid, homocysteine, and IR (P < 0.05) and reduced KCNK3 levels (P = 0.006) compared to controls. ROC analysis showed IR (AUC 0.903), homocysteine (AUC 0.762), and insulin (AUC 0.808) as effective discriminators of PH, whereas KCNK3 had limited diagnostic accuracy (AUC 0.350). Correlation analysis demonstrated positive associations between homocysteine and insulin/glucose, while KCNK3 inversely correlated with renal and metabolic parameters. Subgroup analysis indicated higher urea and glucose in male patients (P < 0.05).

**Conclusion:**

PH is associated with systemic metabolic dysregulation, renal impairment, and molecular alterations, including reduced KCNK3 and elevated homocysteine. Insulin resistance and homocysteine may serve as potential non-invasive biomarkers, highlighting the need for integrated biochemical and molecular assessment in PH management.

## Introduction

Pulmonary hypertension (PH) is characterized by elevated pressures in the pulmonary arteries, ultimately leading to right heart failure and significant mortality if left untreated [1]. Recent revisions to the clinical definition have lowered the threshold for PH to a mean pulmonary artery pressure greater than 20 mmHg [2, 3]. The condition is highly prevalent in other systemic contexts, including chronic kidney disease and heart failure with preserved ejection fraction [4, 5]. Beyond these acquired conditions, PH also manifests as a secondary complication in adults with congenital heart disease and valvular disorders such as aortic regurgitation [5, 6]. While left-sided heart disease remains the most prevalent cause globally, the burden of pulmonary arterial hypertension (PAH) and other forms of PH is heavily influenced by regional disparities, such as the higher prevalence of congenital and connective tissue disease-related cases in Asia and the predominance of infectious and rheumatic heart diseases in developing nations [7, 8, 9]. Recent comprehensive assessments have revealed a shifting epidemiological landscape, with significant increases in PAH-related deaths and disability-adjusted life years observed in high socio-demographic index countries, alongside projections indicating a continued rise in burden, particularly among females and older populations [10].

This disorder is broadly classified, with chronic thromboembolic pulmonary hypertension (CTEPH) standing out as a severe long-term complication of acute pulmonary embolism that remains potentially curable through surgical intervention if identified early [11, 12]. Despite the availability of effective therapies that can improve survival and quality of life, the diagnosis of PH is notoriously difficult and often significantly delayed, with patients frequently enduring symptoms for over a year and consulting multiple physicians before receiving an accurate assessment [11, 13]. This diagnostic inertia is exacerbated by the frequent lack of symptom specificity and the insidious onset of the disease, which can be masked by comorbidities common in elderly populations [14]. Consequently, clinical practice has increasingly turned toward advanced imaging modalities and multidisciplinary evaluation to bridge this gap, utilizing tools such as echocardiography, ventilation-perfusion scintigraphy, and cardiac magnetic resonance imaging to non-invasively assess pulmonary hemodynamics and right ventricular function, which is a critical predictor of patient prognosis [15, 16, 17, 18].

A key molecular player implicated in this disease is the potassium channel subfamily K member 3 (KCNK3), a two-pore domain potassium channel constitutively active at resting membrane potentials, which is crucial for maintaining pulmonary vascular and cardiac homeostasis [19, 20]. Loss-of-function mutations in the KCNK3 gene were identified as the first channelopathy in PAH, and subsequent research has shown that reduced KCNK3 expression or function is a hallmark of both heritable and acquired forms of the disease [21, 22].

Elevated levels of homocysteine have been increasingly recognized as a significant factor in the pathophysiology of both metabolic and cardiovascular diseases, particularly PAH. A growing body of research has investigated the potential of homocysteine as a non-invasive biomarker for assessing risk in patients with PAH, especially those with congenital heart disease (CHD), where it has been observed to be elevated compared to healthy controls [23, 24, 25]. Systemic metabolic dysregulation, particularly insulin resistance (IR), plays important role in the pathogenesis and progression of this condition, with studies by Zhang et al. [26] and Gao et al. [27] exploring the significant association between IR indices and disease severity in both idiopathic and chronic thromboembolic forms of pulmonary hypertension. In this study, creatinine and urea were measured to assess renal dysfunction associated with PAH, while homocysteine was evaluated as a marker of endothelial damage and vascular remodeling. Insulin and glucose were included to reflect metabolic disturbances linked to PAH progression. KCNK3 was investigated due to its key role in pulmonary vascular regulation and its known reduction in PAH, aiming to provide data for the systemic and molecular alterations associated with the PAH.

## Materials and Methods

### Study Design and Setting

This study was designed as a hospital-based case–control study and was carried out at the Respiratory Unit of Al-Sadr Teaching Hospital, Najaf Al-Ashraf, Iraq. The study period extended from January to June 2024. Patients diagnosed with pulmonary hypertension (PH) were compared with apparently healthy individuals serving as a control group to evaluate selected biochemical and molecular biomarkers.

### Study Population

The sample size was determined based on the prevalence of insulin resistance (IR) reported in previous studies of pulmonary arterial hypertension (PAH). Using data from Zamanian et al. [28] (2009), in which 45.7% of PAH females and 21.5% of control females had IR, we calculated the required sample size to detect this difference with 80% power and a two-sided alpha of 0.05. Applying the standard formula for comparison of two proportions, the estimated sample size was approximately 59 participants per group. To account for potential dropouts or missing data, we aimed to recruit at least 60–65 participants in case group; control was considered half in a 2:1 allocation. The case group consisted of patients with confirmed pulmonary hypertension, whose diagnoses were documented in the official medical records of Al-Sadr Teaching Hospital. Diagnosis was based on clinical assessment and echocardiographic findings consistent with pulmonary hypertension. The control group included 30 apparently healthy volunteers, matched for age and sex, with no known history of pulmonary hypertension or other chronic systemic diseases.

### Inclusion and Exclusion Criteria

Inclusion criteria for patients were adults aged 18–70 years with a confirmed diagnosis of pulmonary hypertension and no acute infections at the time of sampling. Exclusion criteria included individuals with chronic kidney disease, liver disease, diabetes mellitus, malignancy, autoimmune disorders, acute inflammatory conditions, or those receiving renal replacement therapy or immunosuppressive drugs. Healthy controls were excluded if they had any history of cardiovascular, metabolic, or renal disease.

### Ethical Considerations

The study protocol was reviewed and approved by the Scientific and Ethical Committee of the College of Science, University of Kufa. All participants provided written informed consent prior to enrollment, and confidentiality of personal data was strictly maintained in accordance with the Declaration of Helsinki.

### Sample Collection and Handling

Height and body weight were measured using standard calibrated instruments, and body mass index (BMI) was calculated as weight (kg) divided by height squared (m²). Age and BMI were recorded for all participants and compared between groups to ensure baseline comparability. Venous blood samples (5 mL) were collected from all participants after overnight fasting (8–10 hours) using sterile disposable syringes. Blood samples were allowed to clot and then centrifuged at 3000 rpm for 10 minutes to obtain serum. Serum samples were aliquoted and stored at −20 °C until biochemical analysis.

### Biochemical Measurements

All blood samples were collected after overnight fasting (8–10 hours). Renal function parameters, including blood urea and serum creatinine, as well as fasting blood glucose levels, were measured using standard spectrophotometric methods with commercially available diagnostic kits, following the manufacturers’ instructions. Serum levels of homocysteine, insulin, and potassium channel subfamily K member 3 (KCNK3) were quantified using enzyme-linked immunosorbent assay (ELISA) kits. Serum troponin (Troponin I) levels were measured using a ELISA kit, following the manufacturer’s instructions, to assess myocardial injury associated with pulmonary hypertension. Insulin resistance was estimated using the Homeostatic Model Assessment of Insulin Resistance (HOMA-IR). Fasting serum insulin (µIU/mL) and fasting blood glucose (mg/dL) levels were measured for all participants. HOMA-IR was calculated as fasting insulin (µIU/mL) × fasting glucose (mg/dL) ÷ 405, with higher values indicating greater insulin resistance.

### Statistical Analysis

Data were processed and tabulated using Microsoft Excel 2016 for calculation of concentrations, means, and standard deviations (SD). Statistical analyses were conducted using SPSS software version 26. Comparisons between patient and control groups were performed using independent sample t-tests. For subgroup analysis, patients with pulmonary hypertension were stratified according to sex. Comparisons between male and female patients were performed using independent sample t-tests, and results were expressed as mean ± SD. Receiver Operating Characteristic (ROC) curve analysis was applied to evaluate the diagnostic performance of the studied biomarkers. Correlations between biochemical parameters were assessed using Pearson’s correlation coefficient. A P-value of < 0.05 was considered statistically significant.

## Results

**Figure-1:**
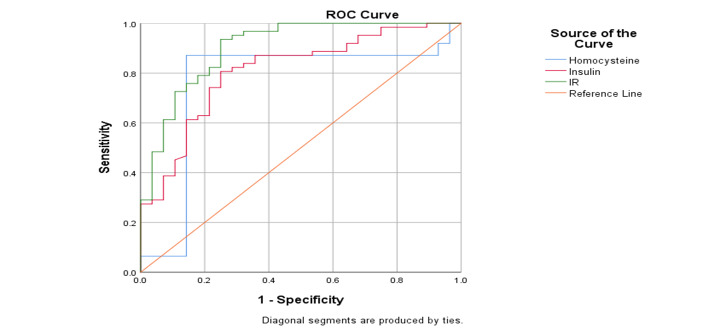
Figure 1. ROC curves of the studied biomarkers (Homocysteine, Insulin, IR and KCNK3)

**Figure-2:**
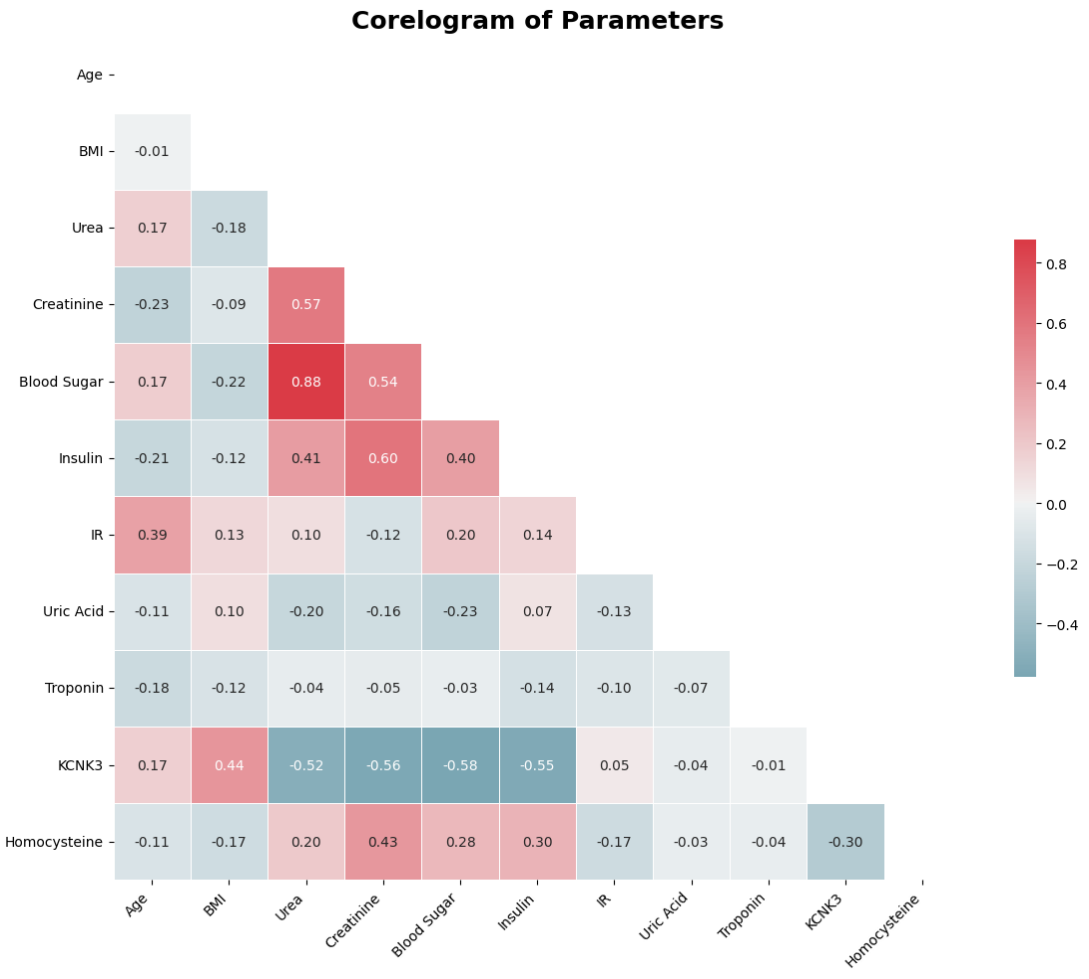
Figure 2. Corelogram showing the correlations between age, BMI, urea, creatinine, blood sugar, insulin, IR, uric acid, troponin, KCNK3, and homocysteine. Positive correlations are shown in red, negative correlations in blue, and the intensity represents the strength of the correlation.

**Table:** Table1. Biochemical and Molecular Parameters in Pulmonary Hypertension Patients and Controls

Parameter	PH Patients (n = 62)	Controls (n = 30)	P-value
Age (years)	58.10 ± 15.44	51.25 ± 13.19	0.369
Gender (male/female, n)	34/28	17/13	0.99
BMI (kg/m²)	25.61 ± 2.54	25.58 ± 2.70	0.950
Urea (mg/dL)	47.74 ± 19.08	33.36 ± 9.05	0.001
Creatinine (mg/dL)	1.43 ± 0.59	0.68 ± 0.15	0.001
Blood Glucose (mg/dL)	210.18 ± 100.91	121.07 ± 30.17	0.001
Insulin (µIU/mL)	174.55 ± 54.72	109.32 ± 43.7	0.001
Uric Acid (mg/dL)	4.48 ± 1.04	4.02 ± 0.91	0.044
Troponin (ng/mL)	0.15 ± 0.08	0.19 ± 0.56	0.653
KCNK3	115.05 ± 8.71	120.59 ± 8.60	0.006
Homocysteine	556.99 ± 77.95	509.80 ± 68.99	0.007

**Table:** Table2. Diagnostic Performance of Biomarkers for Pulmonary Hypertension

Biomarker	Cutoff	Sensitivity (%)	Specificity (%)	AUC	95% CI	P-value
Homocysteine	531.42	87.1	85.7	0.762	0.636–0.889	<0.001
Insulin	133.75	80.6	75.0	0.808	0.712–0.905	<0.001
IR	46.85	82.3	78.6	0.903	0.831–0.974	<0.001
KCNK3	117.77	62.9	60.7	0.350	0.532–0.768	0.023

**Table:** Table3. Comparison of measured parameters between male and female patients with pulmonary hypertension

Parameter	Male (n = 34) M ± SD	Female (n = 28) M ± SD	P-value
Age, years	59.41 ± 14.43	56.50 ± 16.70	0.464
BMI, kg/m²	25.29 ± 2.44	26.00 ± 2.65	0.273
Urea, mg/dl	52.06 ± 17.99	42.50 ± 19.37	0.049*
Creatinine, mg/dl	1.44 ± 0.54	1.42 ± 0.66	0.898
Blood Sugar, mg/dl	241.85 ± 102.54	171.71 ± 85.78	0.005*
Insulin, µIU/ml	165.57 ± 54.91	185.44 ± 53.44	0.157
Uric Acid, mg/dl	4.43 ± 1.20	4.54 ± 0.81	0.680
Troponin, ng/ml	0.16 ± 0.08	0.14 ± 0.08	0.230
KCNK3	115.72 ± 8.75	114.24 ± 8.76	0.509
Homocysteine, µmol/L	553.96 ± 79.02	560.67 ± 77.92	0.739

^*^ P < 0.05 indicates statistical significance.

A total of 92 participants were included, consisting of 62 patients with pulmonary hypertension and 30 healthy controls. There were no significant differences in age, sex distribution, or BMI between the groups (P > 0.05), indicating that the groups were adequately matched. As shown in Table1, patients with PH had significantly higher levels of urea, creatinine, fasting blood glucose, insulin, uric acid, and homocysteine compared with controls. Serum levels of KCNK3 were significantly lower in patients, whereas troponin levels did not differ significantly between groups.

### Diagnostic Performance of Biomarkers

ROC curve analysis (Figure-1) was performed to evaluate the diagnostic performance of the selected biomarkers (Table2). Homocysteine, insulin, and IR demonstrated well to excellent discriminative ability between PH patients and controls, whereas KCNK3 showed limited diagnostic accuracy.

### Subgroup analyses in PAH cases

The correlation analysis among the studied parameters (Table3) revealed several significant relationships. Urea showed a strong positive correlation with creatinine (r = 0.573, P < 0.01) and blood sugar (r = 0.876, P < 0.01), indicating that higher urea levels are associated with elevated glucose and creatinine levels, while it correlated negatively with KCNK3 (r = -0.520, P < 0.01). KCNK3 exhibited significant negative correlations with BMI (r = -0.442, P < 0.01), urea, creatinine, fasting blood sugar, and insulin, suggesting an inverse relationship with metabolic and renal parameters. Homocysteine was positively correlated with fasting blood sugar (r = 0.278, P < 0.05) and insulin (r = 0.301, P < 0.05), indicating a link with glucose-insulin metabolism. Other notable relationships include a positive correlation of age with insulin resistance (IR; r = 0.387, P < 0.01). These correlations are visually summarized in Figure-2, which presents a correlogram of all parameters. Table3 compares measured parameters between male and female patients with pulmonary hypertension. Most parameters did not differ significantly between sexes, including age, BMI, creatinine, insulin, uric acid, troponin, KCNK3, and homocysteine. However, urea levels were significantly higher in male patients (52.06 ± 17.99 mg/dl) than in female patients (42.50 ± 19.37 mg/dl; p = 0.049), and blood sugar was also higher in males (241.85 ± 102.54 mg/dl) compared to females (171.71 ± 85.78 mg/dl; P = 0.005).

## Discussion

While PH is primarily considered a cardiopulmonary disease, our findings reinforce the growing recognition of its systemic effects, particularly on renal function, metabolic homeostasis, and vascular biology. Our study demonstrated significantly elevated serum urea and creatinine levels in patients with PH compared to healthy controls. This finding reflects the interplay between PH and renal dysfunction, a relationship that has been reported in multiple studies [29, 30]. Chronic PH can lead to right heart failure, resulting in reduced cardiac output and renal hypoperfusion. This hypoperfusion activates neurohormonal systems such as the renin-angiotensin-aldosterone system (RAAS), which promotes sodium and water retention, increases vascular resistance, and exacerbates renal injury [31, 32]. Additionally, elevated right atrial pressures in PH cause venous congestion, further impairing renal filtration capacity. The combination of reduced perfusion and renal congestion contributes to the accumulation of nitrogenous waste products, such as urea and creatinine, as observed in our cohort.

Elevated urea and creatinine levels have prognostic significance in PH. Prior studies indicate that increased blood urea nitrogen (BUN) and creatinine correlate with worse survival outcomes, particularly in patients with precapillary PH [31, 32, 33, 34]. Notably, our subgroup analysis revealed that male PH patients had higher urea and fasting blood glucose levels compared to females, suggesting potential sex-related differences in PH severity and associated renal-metabolic dysfunction.

In addition to renal impairment, patients with PH exhibited elevated fasting blood glucose and insulin levels. These findings align with accumulating evidence that PH is associated with systemic metabolic derangements, including insulin resistance (IR) [26, 27]. Chronic hypoxia, systemic inflammation, and endothelial dysfunction, hallmarks of PH, disrupt glucose metabolism and insulin signaling. Insulin resistance may further exacerbate pulmonary vasoconstriction, vascular remodeling, and right ventricular dysfunction [26, 27]. Our results confirm that IR, as estimated by HOMA-IR, has a high discriminatory ability for PH (AUC = 0.903), underscoring its potential as a disease modifier and prognostic biomarker.

Hyperuricemia was also observed in PH patients, albeit modestly. Elevated uric acid levels reflect oxidative stress and tissue hypoxia, processes that are central to PH pathophysiology. Increased serum uric acid has been linked to right heart dysfunction, reduced exercise tolerance, and adverse prognosis [35, 36]. Although no significant sex differences were observed in uric acid levels in our study, its role as a non-invasive marker of PH severity remains relevant.

Our study identified elevated homocysteine and decreased KCNK3 levels in PH patients, supporting their involvement in disease pathogenesis. Homocysteine, a sulfur-containing amino acid, promotes endothelial dysfunction, oxidative stress, inflammation, and vascular smooth muscle proliferation, all critical mechanisms in PH development [23, 24]. Elevated homocysteine impairs nitric oxide bioavailability and contributes to thrombogenesis, thereby increasing pulmonary vascular resistance and cardiac strain. ROC analysis in our study showed that homocysteine has moderate diagnostic discrimination (AUC = 0.762) between PH patients and controls, highlighting its potential utility as a biomarker.

KCNK3 encodes the TASK-1 potassium channel, which is essential for maintaining resting membrane potential in pulmonary artery smooth muscle cells (PASMCs). Loss-of-function mutations or downregulation of KCNK3 lead to PASMC depolarization, increased intracellular calcium, and enhanced vascular remodeling [21, 22]. In our cohort, KCNK3 levels were significantly lower in PH patients, though ROC analysis demonstrated limited diagnostic power (AUC = 0.350). Despite its lower sensitivity, KCNK3 dysfunction remains a key contributor to pulmonary arterial remodeling and right ventricular overload.

## Conclusion

In summary, pulmonary hypertension is associated with multifaceted systemic alterations beyond the pulmonary vasculature. Renal impairment, as indicated by elevated urea and creatinine, reflects reduced renal perfusion, venous congestion, and neurohormonal activation. Metabolic dysfunction, particularly insulin resistance and hyperglycemia, further complicates disease progression. At the molecular level, elevated homocysteine and KCNK3 downregulation contribute to vascular injury, remodeling, and right ventricular overload. These findings highlight the importance of comprehensive assessment of renal, metabolic, and molecular biomarkers in PH patients, and suggest potential therapeutic targets for mitigating disease progression.

## Conflict of Interest

The authors declare no conflict of interest.

## AI Disclosure Statement

During the preparation of this manuscript, the authors used ChatGPT, OpenAI company for language editing, grammar improvement, and liboberry.com for reference management. After its use, the authors thoroughly reviewed, verified, and revised all AI-assisted content to ensure accuracy and originality. The authors take full responsibility for the integrity and final content of the published article.
